# Features of Mobile Diabetes Applications: Review of the Literature and Analysis of Current Applications Compared Against Evidence-Based Guidelines

**DOI:** 10.2196/jmir.1874

**Published:** 2011-09-22

**Authors:** Taridzo Chomutare, Luis Fernandez-Luque, Eirik Årsand, Gunnar Hartvigsen

**Affiliations:** ^1^Norwegian Centre for Integrated Care and TelemedicineUniversity Hospital of North NorwayTromsøNorway; ^2^Medical Informatics & Telemedicine GroupDepartment of Computer ScienceUniversity of TromsøTromsøNorway; ^3^Northern Research InstituteTromsøNorway

**Keywords:** Mobile health (mHealth), diabetes mellitus, blood glucose self-monitoring, social networks, personal health records (PHR), personalized education, diabetes self-management, health informatics

## Abstract

**Background:**

Interest in mobile health (mHealth) applications for self-management of diabetes is growing. In July 2009, we found 60 diabetes applications on iTunes for iPhone; by February 2011 the number had increased by more than 400% to 260. Other mobile platforms reflect a similar trend. Despite the growth, research on both the design and the use of diabetes mHealth applications is scarce. Furthermore, the potential influence of social media on diabetes mHealth applications is largely unexplored.

**Objective:**

Our objective was to study the salient features of mobile applications for diabetes care, in contrast to clinical guideline recommendations for diabetes self-management. These clinical guidelines are published by health authorities or associations such as the National Institute for Health and Clinical Excellence in the United Kingdom and the American Diabetes Association.

**Methods:**

We searched online vendor markets (online stores for Apple iPhone, Google Android, BlackBerry, and Nokia Symbian), journal databases, and gray literature related to diabetes mobile applications. We included applications that featured a component for self-monitoring of blood glucose and excluded applications without English-language user interfaces, as well as those intended exclusively for health care professionals. We surveyed the following features: (1) self-monitoring: (1.1) blood glucose, (1.2) weight, (1.3) physical activity, (1.4) diet, (1.5) insulin and medication, and (1.6) blood pressure, (2) education, (3) disease-related alerts and reminders, (4) integration of social media functions, (5) disease-related data export and communication, and (6) synchronization with personal health record (PHR) systems or patient portals. We then contrasted the prevalence of these features with guideline recommendations.

**Results:**

The search resulted in 973 matches, of which 137 met the selection criteria. The four most prevalent features of the applications available on the online markets (n = 101) were (1) insulin and medication recording, 63 (62%), (2) data export and communication, 61 (60%), (3) diet recording, 47 (47%), and (4) weight management, 43 (43%). From the literature search (n = 26), the most prevalent features were (1) PHR or Web server synchronization, 18 (69%), (2) insulin and medication recording, 17 (65%), (3) diet recording, 17 (65%), and (4) data export and communication, 16 (62%). Interestingly, although clinical guidelines widely refer to the importance of education, this is missing from the top functionalities in both cases.

**Conclusions:**

While a wide selection of mobile applications seems to be available for people with diabetes, this study shows there are obvious gaps between the evidence-based recommendations and the functionality used in study interventions or found in online markets. Current results confirm personalized education as an underrepresented feature in diabetes mobile applications. We found no studies evaluating social media concepts in diabetes self-management on mobile devices, and its potential remains largely unexplored.

## Introduction

Social media and user-friendly mobile devices are one of the most significant recent developments in information and communication technology. As well as being commercially successful and popular, social media hold a potential for interesting new use cases in health care. On the other hand, enhanced usability and pervasiveness of mobile devices have resulted in renewed interest in and development of new requirements for health care applications. Research has consistently shown that diabetes management is one application area [1-5] where mobile devices could enhance the quality of life for people living with chronic illnesses [6].

Although there is now a wide body of literature on the use of mobile devices in self-management of diabetes, present knowledge about good practice in designing integrated health applications seems rather limited. An integrated application provides intermodule and interapplication communication interfaces transparent to the user, resulting in a seamless whole. We have not found research focused on the gaps between the functional requirements (evidence-based recommendations in clinical guidelines) and the functionality available in current tools. Some reviews address the design of user interfaces [7,8] and the effect of mobile applications on health outcomes [9-12], but these studies have not treated functional requirements in much detail.

The conclusion in Farmer and colleagues’ [13] most influential work, a randomized controlled trial involving 93 patients, was that decision-support features were important to realize benefits from blood glucose monitoring. On the other hand, in a UK and Canada review from 2009, Seto et al [14] found no benefits from monitoring blood glucose, but argued for the inclusion of communication with primary care providers [3,15,16] in the design of interventions. In a study involving 30 patients with type 2 diabetes, Faridi et al [17] found no statistically significant improvement in glycosylated hemoglobin with the use of mobile applications compared with standard therapy. It is likely that conflicting outcomes in these and other similar studies are partially due to differences in the design of the mobile tools as interventions. Well-designed mobile tools with decision-support features [13] such as personalized education have demonstrated potential to enhance self-management outcomes [8].

We present an in-depth analysis of the features of diabetes mobile applications. In addition, we contrast the requirements derived from evidence-based recommendations with the functions available in existing interventions. The rationale is to identify gaps and contribute to improving the tools available to the target group. The aim of the analysis is to answer three questions: (1) what functionality is available on the market for diabetes mobile applications?, (2) what gaps exist in relation to the evidence-based recommendations for this target group? and (3) what new use cases from social media could enhance such applications?

## Methods

Our goal was to review as many and as diverse diabetes mobile applications as possible, both in the literature and in commercial markets. Many successful applications do not have any grounding in research, hence our decision to include the online markets and gray literature, where people in general showcase their innovation, often based on personal needs. While the literature typically reflects emerging applications and new trends, the market gives a good indication of mature applications and functionality. Comparing and contrasting the current functionality with recommendations in clinical guidelines constitutes a gap analysis.

### Selection Criteria

The main inclusion criterion was that the application had a self-monitoring of blood glucose (SMBG) component. This inclusion criterion had the potential to preclude relevant applications, but in reality none of the excluded applications had a clear focus on diabetes. We settled on SMBG as the main inclusion criterion in order to filter out applications intended exclusively for medical professionals rather than patients, as well as other general health and lifestyle applications. We excluded applications without English-language user interfaces and those designed exclusively for health care professionals. We also excluded hardware-based solutions geared toward blood glucose tracking or insulin pumps only. Applications with their latest updates or publications prior to 2006 were excluded.

### Search Strategy

The search was based on two main source types. The first source was online journal databases, indexers, and reference lists. We searched for prototypes and work in progress using the search terms “diabetes,” “mobile,” “PDA,” “cell,” “phone,” and “application”. We constructed a search string using both the conjunction “AND” and the disjunction “OR” logical operators (diabetes AND [mobile OR PDA OR cell OR phone OR application]). The search was based on the metadata—that is, title, abstract, and keywords. We targeted both original research papers and review articles indexed by Medline, ScienceDirect, ACM (Association for Computing Machinery) Digital Library, IEEE (Institute of Electrical and Electronics Engineers) Xplore Digital Library, Google Scholar, and DBLP (Digital Bibliography & Library Project) Computer Science Bibliography. The databases reflect the multidisciplinary nature of the research involving both medical and computer science fields. We identified three recent relevant reviews by Årsand et al [18], Tatara et al [7], and Liang et al [4], where we cross-checked descriptions. We also searched the gray literature: technical reports, Internet blogs, and portals.

The second source was online stores for mobile applications, using the search terms “diabetes” and “glucose” with the disjunction “OR” logical operator (diabetes OR glucose). We identified online stores for four leading platforms: Apple iPhone, Google Android, BlackBerry, and Nokia Symbian. We searched these two source types—namely, the online journal databases and the online markets—independently of each other. We searched the online market first, and then subsequently searched the related literature.

### Evaluation and Assessment of Application Features

We analyzed the following features: (1) self-monitoring: (1.1) blood glucose, (1.2) weight, (1.3) physical activity, (1.4) diet, (1.5) insulin and medication, and (1.6) blood pressure), (2) education, (3) disease-related alerts and reminders, (4) integration of social media functions, (5) disease-related data export and communication, and (6) synchronization with personal health record (PHR) systems or patient portals. These features are the result of iterated brainstorming sessions among the coauthors and discussions in focus group meetings with patients and physicians. The emphasis in these sessions was put on translating guideline recommendations into a requirements specification implementable on a mobile phone platform. We created a list with multiple features and in iteration reduced the list to six main features, which we believed had the most potential for enhancing future mobile applications. 

These features are individually quite distinct, but they have the potential to work as an integrated self-management tool. For example, the user could log weight, physical activity, meals, or carbohydrate intake, and have an easy-to-understand visual display to see how they correlate or affect the blood glucose. It should be noted that the “insulin” feature in most applications was part of a customizable “medication” feature for managing other medications as well.

We installed the available applications and recorded the functionality in a spreadsheet (see Multimedia Appendix 1). For those that we were not able to install, we cross-referenced the function descriptions in published articles. We noted whether each of the functions required manual interaction with the user, or whether wired or wireless sensors were used to import data into the application automatically. We then compared the prevalence of features with the recommendations in several clinical guidelines (see Discussion section for references to guidelines). Guideline recommendations can provide a good basis for requirements analysis and specification during the design and development of diabetes applications.

The process of extracting the data presented a major risk of error and uncertainty. For example, the literature is in most instances implicit about the functionality, and it is easy to miss or misunderstand feature descriptions within the text. To avoid potential problems, we enhanced the assessment process with independent verification. While we cannot claim the process we designed is entirely infallible, we avoided likely pitfalls that might otherwise have invalidated our findings. The next paragraph explains the process in some detail.

One author (EÅ) conducted the market and literature search in another related study [18] and another author (TC) conducted another independent search and installation of the selected applications. A third author (LFL) inspected the installation and assessed the feature on randomly sampled applications. The forth author (GH) independently undertook another round of data inspection and verification. Disagreements were settled by discussion and when necessary by redefining categories.

## Results

**Figure 1:**
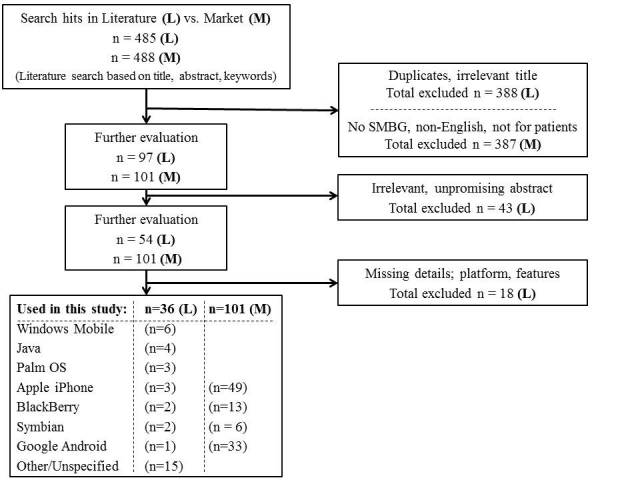
Selection process for online journal databases and online markets (SMBG = self-monitoring of blood glucose).

The breakdown of the search process from online journal databases, gray literature, and online markets is shown in Figure 1. As illustrated in the figure, the total matches were 485 for literature and 488 for online markets, bringing the total matches for this study to 973. We went through a sifting process, with 36 applications from the literature and 101 from the online markets remaining, ending at a total of 137 mobile applications. Of the selected 101 market applications, 40 were available for free. The mean and modal price for the rest of the applications was the equivalent of €2.50 and €1.50, respectively. Of the 40 free applications, 12 had some premium functionality available only at an additional cost.

Some applications were counted multiple times—that is, for each platform or source on which they appeared. Of the total 137 eligible applications, we installed 82 on mobile devices for further analyses and classified the rest as either work in progress or unavailable for installation. Two of the 82 installed applications—namely “Tag-It-Yourself” and “Few Touch”—were from the literature. The former was also available on iPhone and the latter was developed in-house. It is important to note that some studies used commercially available applications but did not explicitly refer to the application names or features, and were thus excluded from this study. 

We labeled many studies as having irrelevant titles; because the search terms included diabetes and mobile, it was common to find purely medical studies on diabetes in medical journals. Our search was based on the title, abstract, or keywords, but even this streamlined search criterion is bound to yield many irrelevant articles. On the other hand, most of the articles that matched the search criteria in information and communication technology journals turned out to contain relevant data for this study. Abstracts that were judged to have low probability of containing relevant data were labeled as unpromising and excluded from this study. 

The features of the mobile applications per mobile platform and source are summarized in Table 1. The figures include the total results from the online stores, journal databases, and gray literature. Explanations of the functionalities are given in Multimedia Appendix 2. The blood glucose monitoring feature is not shown in Table 1 because it is a part of all applications as implied by the selection criteria.

**Table 1:** Numbers and percentages of applications (n = 137) with the respective features of insulin, communication (Comm), diet, physical activity (PA), weight, blood pressure (BP), personal health record (PHR), education (Edu), social media (SM), and alerts

Application	Insulin	Comm	Diet	PA	Weight	BP	PHR	Edu	SM	Alerts
Apple iPhone (n = 49)	35 (71%)	36 (73%)	26 (53%)	17 (35%)	19 (39%)	13 (27%)	7 (14%)	8 (16%)	12 (24%)	7 (14%)
Google Android (n = 33)	19 (58%)	17 (52%)	15 (45%)	10 (30%)	16 (48%)	16 (48%)	7 (21%)	3 (9%)	0 (0%)	0 (0%)
BlackBerry (n = 13)	5 (38%)	6 (46%)	3 (23%)	2 (15%)	5 (38%)	4 (31%)	1 (8%)	2 (15%)	4 (31%)	0 (0%)
Nokia Symbian (n = 6)	3 (50%)	2 (33%)	4 (67%)	4 (67%)	4 (67%)	3 (50%)	2 (33%)	2 (33%)	1 (17%)	1 (17%)
Average for online markets (n = 101)	63 (62%)	61 (60%)	47 (47%)	34 (34%)	43 (43%)	36 (36%)	17 (17%)	16 (16%)	17 (17%)	8 (8%)
Average for literature (n = 26)	17 (65%)	16 (62%)	17 (65%)	15 (58%)	7 (27%)	6 (23%)	18 (69%)	10 (38%)	3 (12%)	7 (27%)
Average for gray literature (n = 10)	9 (90%)	4 (40%)	7 (70%)	5 (50%)	3 (30%)	2 (20%)	5 (50%)	2 (20%)	0 (0%)	1 (10%)
Total weighted average	89 (65%)	81 (59%)	71 (52%)	55 (40%)	53 (39%)	44 (32%)	40 (29%)	27 (20%)	21 (15%)	16 (12%)

Table 1 shows that tools for tracking insulin or other medication were present in 89 (65%) of the applications, although most online market applications did not specify whether the application was meant for type 1 or type 2 diabetes. Just over half of the applications had some form of diet management, either by tracking carbohydrate intake or by providing meal suggestions. Physical activity and weight tracking had 55 (40%) and 53 (39%) applications, respectively. A component for synchronizing with PHRs or Web portals was present in 40 (29%) of the applications. Only seven of the 27 applications with an educational module had personalized education, tips, feedback, or advice. Few applications were sensitive to the age or gender of the users; important specific factors for special user groups such as pregnant women, for example, were largely ignored. Some form of lightweight integration with social media was present in 21 (15%) applications, while 16 (12%) had disease-related reminders. Of the applications randomly sampled for verification checking, 7 (5%) of the 130 features analyzed were in disagreement. None of the disagreements concerned features related to our main findings.

## Discussion

The results in Table 1 are revealing in several ways. Perhaps the most significant outcome apparent in Table 1 is that education is a feature present in only a few diabetes-related mobile applications. Second, we can observe that a small percentage of applications have social media, suggesting that the influence of social media on the development of diabetes mobile applications is so far negligible. Another interesting finding emerging from this study is that most online market applications are based on manual entry of data such as blood glucose levels and weight, while 16 (62%) of the 26 applications found in the literature used wireless (Bluetooth, ZigBee, or Wi-Fi) automatic data acquisition. Wireless sensors are now widely available, but proprietary rights and vendor restrictions hamper their use in some commercial markets (eg, Apple iPhone). Manual data input not only exposes the user to erroneous input, but it can also be a daunting task and may lower compliance [14]. In the remaining subsections, we discuss the details of these results.

### Core Functionality versus Requirements

To discover whether the requirements from clinical guidelines were necessarily met, we turned to what was available on the online markets. However, it was impossible to accurately determine how many of the applications available on the commercial market were used in research or were founded on evidence-based principles. The four most prevalent features can be seen from the data in Table 1. We highlight these features in Table 2 with a slightly different perspective. We omit results from the gray literature in the table because of its potential to obfuscate important elements that the table illustrates.

**Table 2:** The most prevalent features (n = 137 applications) on the online markets versus in the literature

Order	Online stores	Literature	Overall weighted prevalence
1.	Insulin, 63 (62%)	Personal health record, 18 (69%)	Insulin, 89 (65%)
2.	Communicating, 61 (60%)	Insulin, 17 (65%)	Communication, 81 (59%)
3.	Diet, 47 (47%)	Diet, 17 (65%)	Diet, 71 (52%)
4.	Weight, 43 (43%)	Communication, 16 (62%)	Physical activity, 55 (40%)

The results obtained from online journals and markets are compared and weighted in Table 2. From the data, we can see that most applications used in the literature integrated with a PHR, despite the intricacies associated with PHR integration. Outside well-controlled research, however, it is typically more difficult to offer PHR functionalities for facilitating collaborative care. In addition to application development challenges, more threatening are legislative and organizational barriers related to communicating patient data. The existing restrictive environment has hampered adoption and discouraged potential innovators such as Google Health. In Table 2, the PHR feature disappears from the weighted list because of the sample size imbalance between the market (n = 101) and the literature (n = 26).

It is easy to see that the rankings from Table 2 are biased toward patients with more severe illness, where the patients use insulin. This is unexpected because between 90% and 95% of people with diabetes have type 2 diabetes. Most people with type 2 diabetes do not use insulin, but rather oral medication and lifestyle changes such as diet and physical activity. This bias toward insulin-based solutions initially seems counterintuitive in light of the stated statistical data, but the underlying premise is based on known results [19,20]. Although there is still much disagreement among researchers, evidence of the benefit of intensive blood glucose monitoring for patients not using insulin seems rather weak [19,20,21].

Recent advances reflected in these clinical guidelines [22-24] recommend the following features (in random order) as part of important variables for diabetes self-management:

1. Education and personalized feedback;
2. Diet management;
3. Weight management;
4. Physical activity;
5. Communication and patient monitoring by primary care providers;
6. Insulin and medication management;
7. Other therapeutics (foot, eye care);
8. Psychosocial care;
9. Immunization;
10. Complication management.

**Figure 2:**
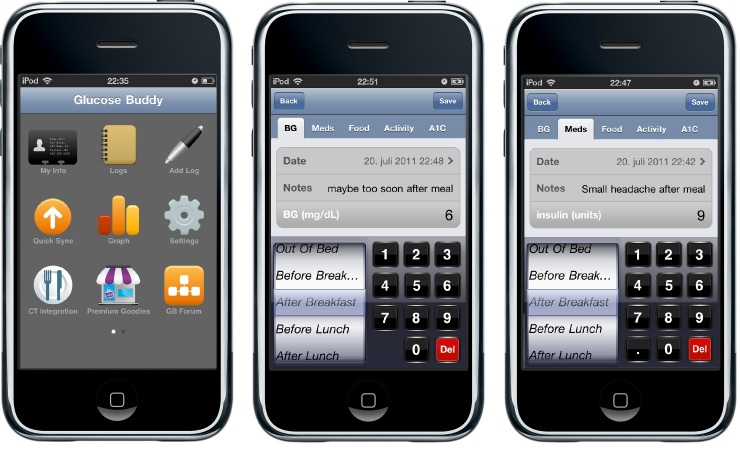
Glucose Buddy iPhone application screenshots showing the main menu (left), blood glucose logging (center), and medication logging (right).

**Figure 3:**
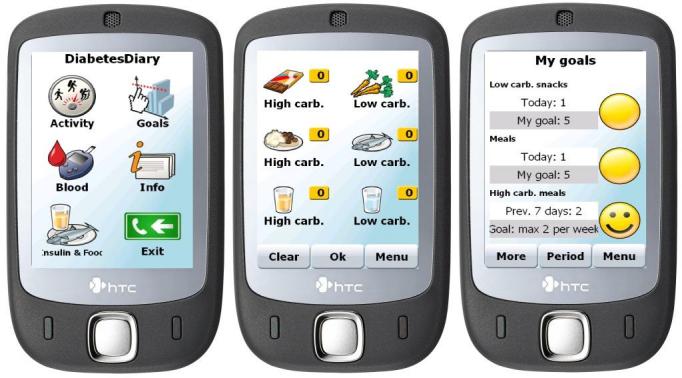
Few Touch Windows Mobile application screenshots showing the main menu (left), food registration (center), and feedback on diet goals (right).

It is important to note that current applications meet the functional requirements list only partially. Some of the features are shown in the screenshots of two sampled applications in Figure 2 and Figure 3. Figure 2 shows an iPhone application, Glucose Buddy. The application has a touch-sensitive interface and can also be installed on compatible mobile devices such as Apple iPods. Figure 3 shows a Windows Mobile application, Few Touch. This application also has a touch-sensitive interface and can be installed only on devices with the Windows Mobile operating system. The applications in both Figure 2 and Figure 3 are platform specific, as are most mobile applications.

### Classification of Functionality

**Figure 4:**
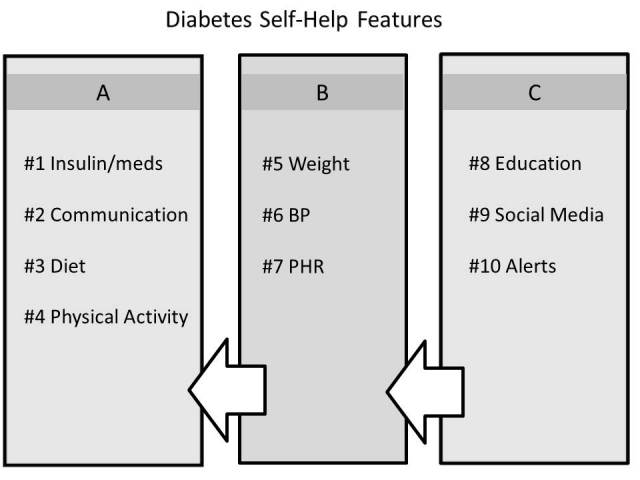
Arbitrary classification of functionality based on prevalence in the surveyed mobile applications (BP = blood pressure; PHR = personal health record).

Figure 4 illustrates an arbitrary classification of the surveyed mobile applications on the basis of prevalence. Class A comprises the four major features. Class B functionality comprises weight management, blood pressure monitoring, and PHR integration. These have a significantly higher prevalence than the class C features, which comprise education, social media integration, and alerts. In the future, we expect the ideal application to have all the features available as part of the core application, resulting in an integrated, feature-rich, and adaptive system. The presented classification may be useful for application developers and intervention designers when considering the features to implement. In addition, the classification is intended to draw attention to the least prevalent and less well-studied features.

Although the Action to Control Cardiovascular Risk in Diabetes (ACCORD) study group [25] found no significant benefits of intensive antihypertensive therapy for patients with type 2 diabetes at risk of cardiovascular events, blood pressure monitoring is likely to be part of class A functionality as wireless blood pressure sensors for home and personal use become more ubiquitous. Weight management is important for overweight and obese patients with diabetes. However, a good percentage of the patients do not struggle with weight; therefore, weight management seems rationally placed in class B. A PHR integration feature was implemented in most scientific studies, but because it was not available in online markets where more applications were sampled, it remained in class B.

It is somewhat surprising that education is conspicuously underrepresented, even though clinical guidelines suggest it belongs in class A. Structured and personalized education and actionable feedback are widely suggested as the missing link for people with diabetes who do not use insulin. It is not entirely clear why social media and alerts had very low prevalence, but it could partially be because they are difficult to implement. In the succeeding two subsections we discuss education and social media features in some detail.

### Personalized Structured Education: The Missing Link

Current results reveal something completely unexpected: only 27 (20%) of the applications had an education module, and only 7 (26%) of these met our criteria for personalized education or feedback. A recent study [26] showed that, although Internet health information is growing rapidly, the average person lacks the skills for finding and using the health information strategically for his or her benefit. For people with diabetes who do not use insulin, personalized structured education may be the missing link to deriving benefits from SMBG. Some reviews have supported the view that SMBG does not benefit patients who are not using insulin [19,20,21,27]—having the main end point as glycosylated hemoglobin. In contrast, Polonsky et al [28] argue that previous research has not considered structured SMBG with proper education [28,29], where participants are empowered with actionable knowledge on how to deal with different circumstances related to blood glucose variability. Clar et al [19] also agree with the view that proper actionable knowledge will result in SMBG benefits for people not using insulin or oral agents [19,30,31]. Existing accounts fail to resolve the contradiction between the two views. Larger clinical trials with well-defined interventions are needed to provide definitive evidence.

Nonetheless, in terms of design and development of personalized education modules, the task is challenging and the research field is still undeveloped. There is considerable scope for personalization because the mobile applications have access to some data about the users and their health status. However, using these user data for personalizing education is obviously not trivial. Personalizing health education is a rich and interesting field of inquiry that deserves urgent attention.

### Social Media: Emerging Use Cases

In a recent survey, Chen [32] showed the importance of social aspects and experience-sharing among people with diabetes. Chen’s findings underscore the importance of individuality and the need for tailored social interactions, which resonate with the concept of “PatientsLikeMe” [33,34], which has recently received enormous attention.

Findings from this study suggest very little influence of social media on current diabetes mobile applications. Most applications that claim to include social media features only provide a link to their groups in well-known social networking sites such as Facebook and Twitter. Some applications also provide the user with an account to a forum. However, there are no functional links or integration between information in the mobile application and the social media application. For instance, it is not easy to share graphs and data in the mobile applications with friends or relatives in social networks.

Integrating mobile applications with social media presents an opportunity for finding similar users and communities in a dynamic fashion. The health data that these applications store can be used to model the health status, which can then be used to find peers. These new techniques can be applied to create new personalized features, such as recommender systems of educational content [35,36]. Relying on peers for practical support and not entirely on primary health care may lessen the strain on health care resources.

Some applications, however, do synchronize their data with an online portal or PHR automatically. Despite previous research having reported the benefits of patient-accessible electronic health records [37], the rate of adoption of PHR is less than initially expected, partially because of usability issues [38]. Parts of the PHRs and portals can be shared with friends, family members, or physicians. Securely integrating health data and social media holds a potential for enhanced peer support. In the project TuAnalyze [39], researchers from Harvard Medical School have created an application for the diabetes online community TuDiabetes that gathers real time information about different health aspects. That social application is based on the PHR platform Indivo, and is a good example of a successful integration agenda.

### Limitations

Many of the applications found outside the official online stores were not available for installation. As a result, some of the functionality was recorded from only the description or from published articles. Often there are discrepancies between the text description and the actual features, and some functionality is not apparent until the application is installed and tested. In research articles, some authors are not clear about whether any of the work they describe has been done. For example, Buranarach et al [40] and Chang et al [41] discuss Web-based portals for diabetes self-management, but we could not find the portals or verify some of the reported claims. We therefore had to cross-reference the descriptions using three independent review articles. The scope of this study did not include analysis of patient privacy and security issues. Few studies addressed this issue, which is a field in definite need of research.

Another limitation may be that the main inclusion criterion of selecting only applications with an SMBG feature had the potential to preclude other potentially relevant applications. However, in practice, most of the initial 973 search hits included general health information and health news aggregators. Also included in the initial hits were applications for managing physical activity, diet, or weight, but without special regard to diabetes. Most of the more general lifestyle applications such as diet management have a wider audience and are geared toward keeping healthy and fit. In order to maintain some level of focus, blood glucose tracking appeared to be an attractive inclusion criterion for diabetes mobile health applications, and also seemed to be a generally accepted core feature among developers of diabetes applications. 

Since this study only analyzed the availability of applications and their features, it lacks information about the users. One cannot easily make generalizations to populations, since smartphones are not equally used among people of different socioeconomic status and age groups. In addition, it is very hard to know whether the applications are mostly used in developing countries where the available mobile technology may be less sophisticated.

### Conclusions

A main finding from this review is that a critical feature strongly recommended by clinical guidelines—namely, personalized education—is not assimilated in current applications. Polonsky et al [28] and Klonoff [42] argue that studies fail to show SMBG benefits for patients who are not taking insulin because the studies have not integrated well-structured education as part of the intervention, and our results seem to support their premise.

The other major finding emerging from this study is that potentially interesting new use cases from social media are largely undeveloped. Although there is some evidence of the use of PHR in augmenting social engagement with peers, we found that the concept is still seldom recognized in the surveyed applications.

The impact of specific application features on clinical outcomes is not easy to determine, but current findings enhance our understanding of how the lack of some designated core features may influence clinical outcomes. The presented findings contribute evidence that shows personalized education and decision-support features not being integrated in most current blood glucose monitoring interventions, despite the evidence-based recommendations and requirement for them.

## Multimedia Appendix 1

Mobile Apps - Data Supplement.

## Multimedia Appendix 2

The different functions and features explained.

## Abbreviations

ACCORD: Action to Control Cardiovascular Risk in Diabetes
mHealth: mobile health
PHR: personal health record
SMBG: self-monitoring of blood glucose
