# Supplementary material for: Features of Mobile Diabetes Applications: Review of the Literature and Analysis of Current Applications Compared Against Evidence-Based Guidelines
Source: J Med Internet Res. 2011 Sep 22;13(3):e65. doi: 10.2196/jmir.1874 (PMC3222161; doi:10.2196/jmir.1874)
Supplement: Supplementary file 2 [file jmir_v13i3e65_app2.pdf]

#	Function	Input / Output	Summary
1.1	Blood Glucose (self-monitoring)	Blood glucose values, pre-/postprandial tagging	User enters values and can view graphs, with low, high and normal ranges well demarcated. Glucometers are used.
1.2	Weight (self-monitoring)	Weight values (kg, lbs.)	User enters values and can view graphs, can also determine BMI if height is supplied. Wireless weight scales used by some.
1.3	Physical Activity (self-monitoring)	Physical Activity type, intensity, duration	Highly manual task. User has to choose activity type, intensity and duration. Varied motivational output graphs, smileys, etc.
1.4	Diet (self-monitoring)	Food eaten, carbohydrate estimates	Highly manual task. User has to estimate carbohydrates or navigate a comprehensive food menu. Visual summaries, advice given.
1.5	Insulin (self-monitoring)	Insulin type, amount, pre-/postprandial tagging	User enters values, views graphs
1.6	Blood Pressure (self-monitoring)	Blood Pressure values	User enters values, views graphs. Some wireless meters used.
2	Education	Tips, feedback, diabetes health information	Most applications linked to another application for educational material or used web links. Some had decision support and personalized tips and feedback.
3	Alerts	Diabetes relevant reminders	User enters reminders manually, and also receives reminders for postprandial testing. Some had automatic tailored alerts.
4	Social Media	Social data	Some applications have a link to their social networking websites or forums. No seamless functional links.
5	Communication	Email, import/export csv, pictures, graphs	Most applications allowed exporting data to a spreadsheet or emailing data or pictures
6	PHR	Personal Health Record system data	Applications synchronize with the PHR or online patient/doctor portal. The integration was seamless in most cases.
